# Fungal Iron Availability during Deep Seated Candidiasis Is Defined by a Complex Interplay Involving Systemic and Local Events

**DOI:** 10.1371/journal.ppat.1003676

**Published:** 2013-10-17

**Authors:** Joanna Potrykus, David Stead, Donna M. MacCallum, Dagmar S. Urgast, Andrea Raab, Nico van Rooijen, Jörg Feldmann, Alistair J. P. Brown

**Affiliations:** 1 Aberdeen Fungal Group, School of Medical Sciences, Institute of Medical Sciences, University of Aberdeen, Foresterhill, Aberdeen, United Kingdom; 2 Trace Element Speciation Laboratory, Department of Chemistry, College of Physical Science, University of Aberdeen, Meston Walk, Aberdeen, United Kingdom; 3 Department of Molecular Cell Biology, VU University Medical Center, Amsterdam, Netherlands; David Geffen School of Medicine at University of California Los Angeles, United States of America

## Abstract

Nutritional immunity – the withholding of nutrients by the host – has long been recognised as an important factor that shapes bacterial-host interactions. However, the dynamics of nutrient availability within local host niches during fungal infection are poorly defined. We have combined laser ablation-inductively coupled plasma mass spectrometry (LA-ICP MS), MALDI imaging and immunohistochemistry with microtranscriptomics to examine iron homeostasis in the host and pathogen in the murine model of systemic candidiasis. Dramatic changes in the renal iron landscape occur during disease progression. The infection perturbs global iron homeostasis in the host leading to iron accumulation in the renal medulla. Paradoxically, this is accompanied by nutritional immunity in the renal cortex as iron exclusion zones emerge locally around fungal lesions. These exclusion zones correlate with immune infiltrates and haem oxygenase 1-expressing host cells. This local nutritional immunity decreases iron availability, leading to a switch in iron acquisition mechanisms within mature fungal lesions, as revealed by laser capture microdissection and qRT-PCR analyses. Therefore, a complex interplay of systemic and local events influences iron homeostasis and pathogen-host dynamics during disease progression.

## Author Summary

Microbial pathogens must assimilate essential micronutrients to establish infections. During bacterial infection, mammals limit the availability of micronutrients to inhibit the growth of the pathogen – a phenomenon termed ‘nutrient immunity.’ Nutrient immunity has not been examined during disseminated candidiasis. Yet micronutrient assimilation, and iron assimilation in particular, is required for fungal virulence, and life-threatening disseminated fungal infections are recognised as a major medical threat for patients with compromised immune systems. We show that nutrient immunity operates during disseminated *Candida albicans* infections in mice. Over time immune cells congregate around the fungal lesions in the kidney cortex, driving nutrient immunity and reducing iron availability for the pathogen. The fungus responds by tuning its iron assimilation strategies to the reduced iron levels. Paradoxically, iron levels increase in other parts of the kidney as *Candida* infections progress. We show that the fungal infection disturbs global iron homeostasis in the host by perturbing red blood cell recycling in the spleen and this is associated with increased iron storage in the kidney medulla. Therefore, fungal infection exerts system-wide effects upon iron homeostasis in the mammalian host, whilst triggering local nutrient immunity to limit the infection.

## Introduction

Iron is one of the most abundant elements on Earth and, as an important cofactor in metabolic conversions and a mediator of redox reactions, iron is an essential micronutrient for most organisms. On the other hand, excess iron is toxic mediating the formation of potentially deleterious free radicals [1]. Therefore the cellular acquisition, storage and release of iron are tightly controlled. Iron is usually present in the insoluble ferric form (Fe(III)) at neutral pH [1]. However, most iron in the mammalian body is found in the bloodstream bound to haemoglobin. It is estimated that 2×10^11^ erythrocytes are turned over every day in the adult human [2], but dietary iron is insufficient to replenish the potential losses of this red blood cell senescence. Therefore, the iron from senescent red blood cells is recycled by macrophages of the reticulo-endothelial system [3].

During bacterial infection, iron availability is tightly regulated in the host with a view to limiting the availability of this micronutrient, which is essential for the pathogen [4], [5], [6]. This phenomenon has been dubbed ‘nutritional immunity’ [7], [8]. Iron availability is regulated mainly through the hepcidin-ferroportin axis. The liver-derived peptide hormone hepcidin binds to the only characterised mammalian iron exporter, ferroportin, causing its internalisation and degradation, thereby leading to a drop in extracellular iron levels [3]. Modulation of hepcidin levels affects cellular iron pools to minimise pathogenic outgrowth [4]. Furthermore, host iron homeostasis interacts with the immune defences. For example, IL-6 induces hepcidin expression, while increased intracellular iron concentration activates NF-ΚΒ [3], [9]. Iron is also required for the oxidative burst that enables phagocytic killing of pathogens [10]. In turn, iron partitioning within the host influences microbial niche tropism [6]. The impact of iron during bacterial infection is further illustrated by the observation that iron administration can increase mortality rates in infected patients [11], [12].

Fungal pathogens represent an increasingly important clinical problem that affects the lives of millions of individuals worldwide and imposes an economic burden representing billions of pounds [13]. Evidence is mounting that iron availability in the host also has a significant impact upon fungal pathogens. The inactivation of iron assimilation mechanisms attenuates the virulence of major fungal pathogens such as *Candida albicans* and *Cryptococcus neoformans* [14], [15]. The link between iron availability and disseminated mucormycosis is well established [16]. Similarly, iron overload exacerbates the progression of candidiasis in mice [17]. Nevertheless, the ability of the host to limit the progression of fungal infections via iron nutritional immunity has not been explored. Aside from reports of increased hepcidin levels during fungal infection [18], little is known about the *in situ* competition between the fungal pathogen and host for this essential micronutrient.

*Candida albicans* is a major fungal pathogen of humans. This yeast is a frequent cause of mucosal infection (thrush), and a common cause of nosocomial bloodstream infections in severely immunocompromised hosts, which are associated with 40% mortality [13], [19]. Most individuals carry *C. albicans* as a relatively harmless commensal organism in their microflora, and the transition from commensalism to pathogenicity can be difficult to detect. Therefore the early and accurate diagnosis of systemic candidiasis is particularly challenging. Furthermore, new antifungal therapies are being sought to complement the current armoury of clinically useful antifungal drugs [13]. Therefore significant efforts are being invested in the study of fungal metabolic versatility with a view to identifying novel therapeutic approaches [20], . Blocking either reductive iron assimilation (via *FTR1* deletion) [24] or haem-iron acquisition (via *HMX1* deletion) [25] attenuates the virulence of *C. albicans*. Therefore, the inhibition of fungal iron assimilation presents a potential therapeutic strategy. Given the dynamic nature of fungus-host interactions [26], our aim was to characterise the spatio-temporal distribution of iron in host niches during disease progression and the accompanying molecular responses of the invading fungus *in situ*. We reveal a complex interplay between systemic and localised events, which govern fungal iron availability during disseminated candidiasis. We also show that the fungus adapts to these changes in micronutrient availability by adjusting its iron acquisition strategies during disease progression.

## Results

### Systemic candidiasis affects global iron homeostasis in the host

**Figure 1:**
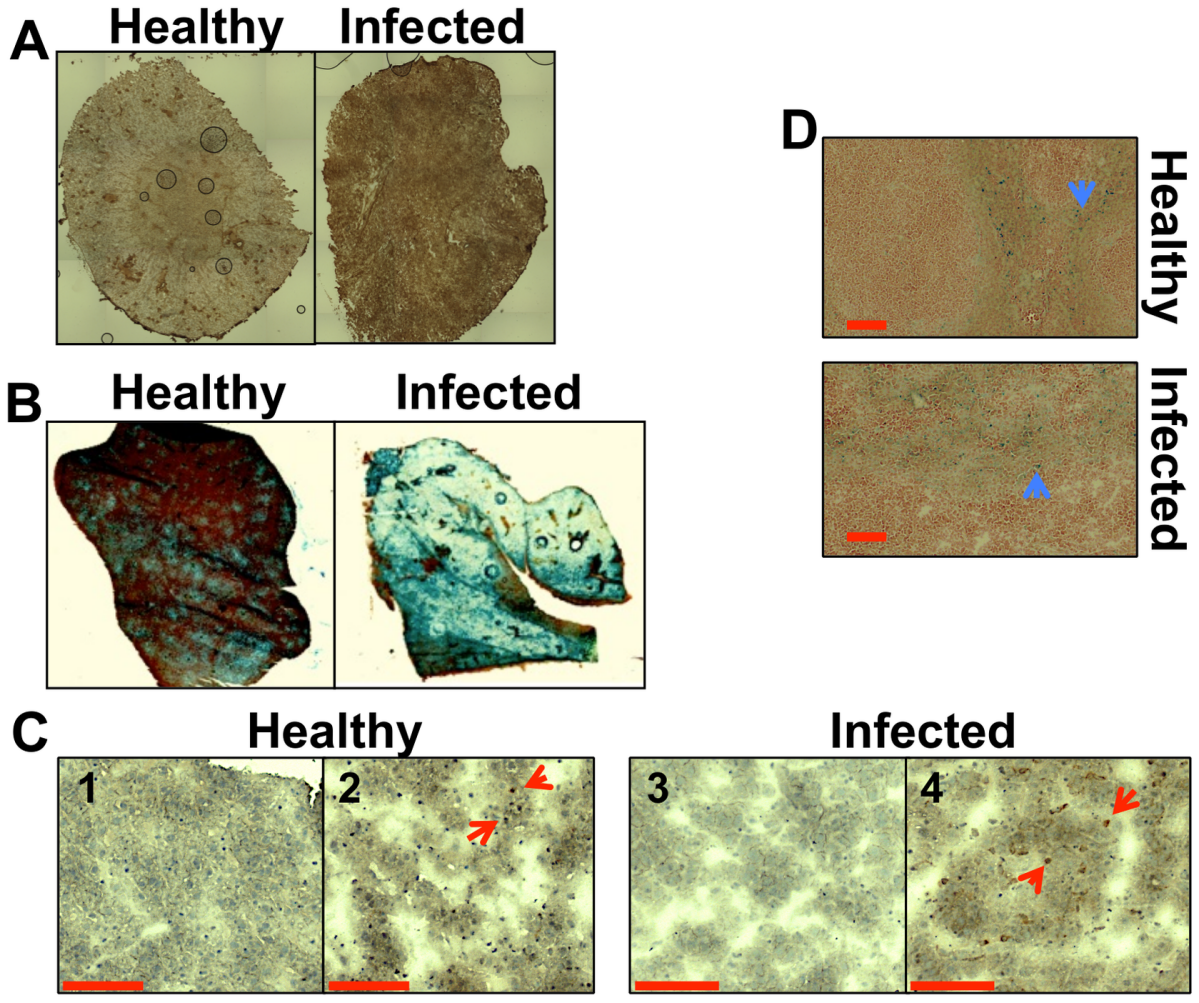
Systemic candidiasis affects host iron homeostasis. A. Immunohistochemical detection of hepcidin in kidneys of healthy (right) and infected (left) animals. **B.** Perls staining of non-haem iron in livers of healthy and infected animals. The intensity of the blue stain is proportional to the amount of hepatic non-haem iron. **C.** Immunohistochemical analysis of Kupffer cells in mouse liver with anti-HO1 antibodies. The red arrowheads indicate hepatic Kupffer cells stained dark brown with anti-HO-1 antibodies. 1C1& 1C3 represent negative controls lacking the primary antibody. **D.** Perls staining of non-haem iron deposits in spleens of healthy and infected animals. The blue arrowheads highlight splenic non-haem iron deposits. *C. albicans* SC5314 infections were conducted in BALB/c mice and all images are representative of at least three separate biological replicates. Size bars correspond to 100 µm. Experimental details can be found in Materials and Methods.

Hepcidin is a hepatic hormone that exerts its effects at a system-wide level, regulating the saturation of iron pools within the body [3], [4]. Disseminated candidiasis is accompanied by increased hepatic hepcidin levels [18]. We examined renal hepcidin levels during systemic candidiasis, revealing elevated hepcidin in the kidneys of infected mice (Fig. 1A). Furthermore, histochemical analyses revealed that hepatic iron storage was more pronounced in animals with candidiasis, when compared to healthy controls (Fig. 1B). This correlated with an increase in the amount of the haem iron-extracting enzyme, haem oxygenase 1 (HO-1) in these organs (Fig. 1C). At the same time, the distribution of iron in the spleen remained relatively unchanged (Fig. 1D). We conclude that global iron homeostasis in the host is perturbed during systemic candidiasis.

### Disseminated *C. albicans* infection causes abnormal iron loading and redistribution in the kidney

**Figure 2:**
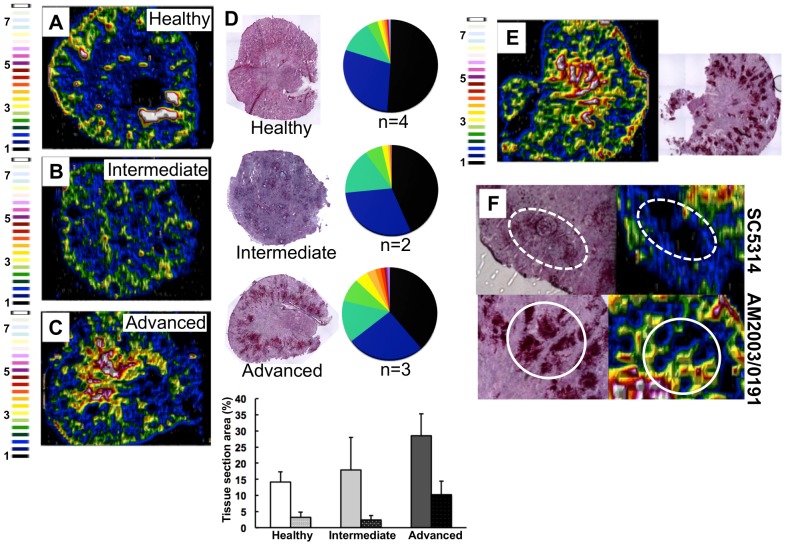
*C. albicans* infection is accompanied by dramatic changes in the renal iron landscape. LA-ICP MS mapping of iron distribution in transverse mouse kidney sections. Normalised ^56^Fe/^13^C ratios are presented, with the colour scale indicating fold increases in signal intensities relative to background. As the infection progresses, iron loading increases and the iron becomes redistributed from the cortex of healthy kidneys (**A**), to the medulla in intermediate (**B**) and advanced infections (**C**). Histology insets (**D**) are representative of healthy, intermediate and advanced infections, and correspond to the tissues imaged in panels **A**–**C**. The pie charts in (**D**) present the percentage total tissue area with a given ^56^Fe/^13^C intensity and the colour scale represents increments of 0.5-fold intensity changes from background (black) to 8-fold increase (white). In the bar chart (**D**) bars correspond to the percentage surface area with normalized ^56^Fe/^13^C intensity ≥2-fold (left, light coloured bars) and ≥3-fold above background (right, dark coloured bars): error bars, standard deviations from the mean; *n*, number of biological replicates. The effects observed with the virulent *C albicans* isolate SC5314 (epidemiological clade 1) are replicated with a different clinical isolate AM2003/0191(clade 2) [30] (panels **E** and **F**, bottom). Infections with *C. albicans* SC5314 stimulate significant immune infiltrates (**F**, top, dotted line), while AM2003/0191 elicits minimal immune infiltrates (**F**, bottom, solid line).

We focused on the organ with the highest fungal burdens in the classical murine model of systemic candidiasis [27], and mapped renal iron (^56^Fe) distributions. Mice were infected with the virulent clinical *C. albicans* isolate SC5314 [28], and iron distributions were mapped across kidney sections from animals at various stages of infection (Fig. 2) using laser ablation-inductively coupled plasma mass spectrometry (LA-ICP MS) [7], [29]. Renal fungal burdens increased during disease progression, and this correlated with a redistribution of iron from the cortex in healthy control animals (Fig. 2A) to the medulla and medullary pelvis in infected animals (Figs. 2B &2C).

To quantify the iron loading in diseased kidneys, ^56^Fe intensities were normalized against the corresponding ^13^C intensities (a proxy for biomass) across the same tissue sections [29]. For animals with advanced candidiasis, regions of high iron loading (^56^Fe/^13^C ratio at least twice the background) accounted for 30% of the total kidney section area, which was double that for healthy controls (Fig. 2D). When the cut-off for normalized ^56^Fe/^13^C signal intensities was raised to three times background, a three-fold difference in intensity was observed in iron loaded areas between infected and uninfected kidneys (∼10% versus ∼3%) (Fig. 2D). This represented an average increase in iron levels from 95±19 µg/g in the healthy renal medulla, to 195±26 µg/g in an infected kidney medulla, respectively. Therefore, *C. albicans* infection affected the loading as well as the distribution of iron in infected kidneys.

This redistribution of renal iron was replicated during infections with a second *C. albicans* clinical isolate from a different epidemiological clade [30] (AM2003/0191: Fig. 2E). Furthermore, the degree of cortical-to-medullary redistribution of renal iron corresponded to the size and number of lesions detected in the kidney (cf. Fig. 2E, more than 40 lesions 250–300 µm in diameter; and Fig.S1, ∼15 lesions 50–100 µm in diameter). In addition, LA-ICP MS mapping of renal iron distributions in mice infected with *C. albicans* strains that display reduced virulence indicated that a sustained *C. albicans* infection must be established to trigger iron accumulation in the renal medulla (Fig. S2).

This iron redistribution was accompanied by the establishment of iron exclusion zones in the renal cortex that correlated with the positions of fungal lesions (Fig. 2F). Furthermore, the size of the iron exclusion zones around fungal lesions correlated with the mass of immune infiltrates encompassing these lesions (Fig. 2F). This observation is consistent with previous studies pertaining to the active role of immune infiltrates in nutritional immunity [7]. It was recently shown that, during bacterial infection, neutrophil infiltration drives the redistribution of manganese and zinc in infected tissues around the infection foci [7].

### Redistribution of globin and haem in the kidney accompanies *C. albicans* infection

**Figure 3:**
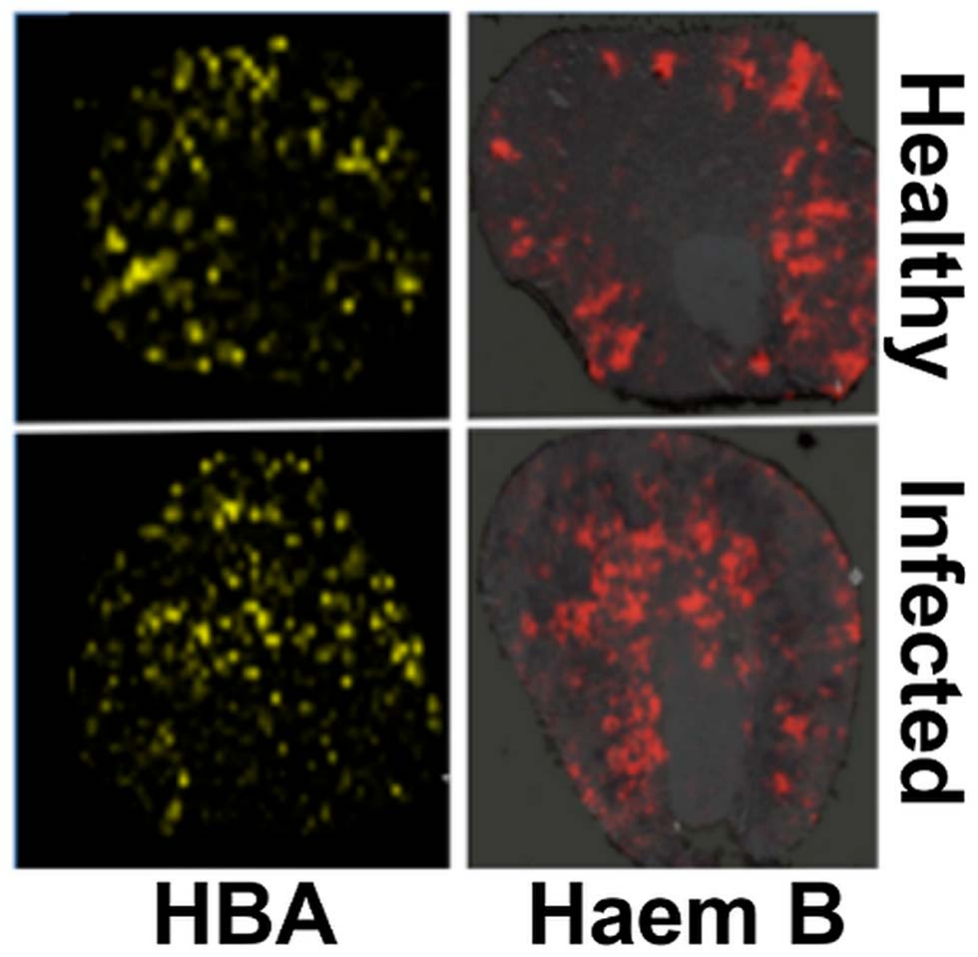
Spatial distributions of HBA and haem mirror ^56^Fe distributions in healthy and infected kidneys. Tissue sections were analysed with MALDI IMS to map the distributions of HBA and haem B (see Materials and Methods, and Supporting Material). Similarly to iron distribution (Fig. 2), in the healthy tissue (top panels) both HBA and haem are distributed to the cortex, whereas in kidneys obtained from animals with advanced infection (bottom panels) they are medullary. Transverse kidney sections sequential to those presented in Fig. 2A (‘Healthy’) and 2C (‘Infected’), respectively, are shown. The images are representative of at least three biological replicates.

Native MALDI MS imaging (MALDI IMS) [31], [32] was used to examine the kidney proteome *in situ*. This revealed an ion of *m*/*z* 14981 that displayed significant differences in its spatial distribution across healthy versus infected kidneys (Fig. S3). The identity of this polypeptide was murine haemoglobin alpha (HBA) (gi|122441) as revealed by LC-MS/MS in conjunction with MALDI-TOF MS (see Materials and Methods). The spatial distribution of HBA replicated that of iron, in that HBA was predominantly located in the renal cortex of healthy animals and elevated in the renal medulla of mice with advanced candidiasis (Fig. 3, Fig. S4). Furthermore, the distribution of a small species of *m/z* 617.6, which was identified as haemoglobin haem B based on its LIFT fragmentation spectrum [33] (Fig. S5C), also reflected iron distribution patterns during infection (Fig. 3, Fig. S4).

Comparisons of replicate MALDI TOF spectra of tryptic peptides from healthy and infected kidneys confirmed that HBA was significantly overrepresented in the renal proteomes of infected animals relative to the healthy controls. Indeed, the HBA peptides IGGHGAEYGAEALER (ion *m/z* 1529.73) and TYFPHFDVSHGSAQV (ion *m/z* 1819.88) were amongst the most discriminatory peptides between the infected and uninfected renal proteomes (Fig. S5, Table S1). In contrast, the spectral abundance of tryptic peptides derived from haemoglobin beta subunits 1 and 2 (HBB) (gi|122513; gi|17647499) did not differ significantly between the healthy and infected states (Fig. S5; Table S1).

### Renal iron redistribution during *C. albicans* infection is regulated by host proteins

**Figure 4:**
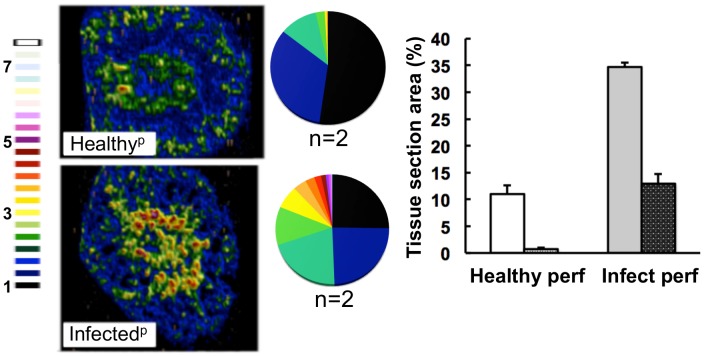
The medullary iron accumulated in advanced candidiasis is associated with renal tissue. LA-ICP MS was used to map iron (^56^Fe) distribution in transverse kidney sections after rough perfusion of the organs with saline. Saline perfusion displaced most iron from healthy tissue (‘Healthy^p^’, **top**), in contrast to infected tissue (‘Infected^p^’, **bottom**), suggesting a tissue-associated pool of iron that accompanies systemic *Candida* infection. Pie charts present the percentage total tissue area with a given ^56^Fe/^13^C intensity and the colour scale represents increments of 0.5-fold intensity changes from background (black) to 8-fold increase (white). In the bar chart, bars correspond to the percentage surface area with normalized ^56^Fe/^13^C intensity ≥2-fold (left, light coloured bars) and ≥3-fold above background (right, dark coloured bars): error bars, standard deviations from the mean; *n*, number of biological replicates.

To test whether the elevated iron levels in the kidney were associated with renal tissue or the vasculature of this organ, kidneys were perfused with saline before LA-ICP MS imaging. This procedure depleted the cortical iron from healthy kidneys suggesting that this iron was primarily associated with red blood cells. In contrast, perfusion failed to displace the medullary iron from infected tissue (Fig. 4), suggesting that during candidiasis, excess iron was deposited in the medulla of infected kidneys.

Iron accumulation in the kidney is often associated with severe tissue injury, haemolysis and haematuria [3]. It has been reported that animals with disseminated candidiasis succumb to progressive sepsis and can develop severe renal failure [34]. However, several observations indicated that the renal accumulation of iron during systemic candidiasis was not caused by kidney injury and haemolysis in our infection model. Firstly, histological analyses revealed no pronounced changes in kidney architecture. Secondly, changes in renal iron distribution were apparent early in the infection process before large fungal lesions had developed and significant tissue damage was incurred (Fig. S1). Thirdly, the differential accumulation of alpha and beta globins (HBA and HBB) was inconsistent with internal haemorrhage. Fourthly, only sporadic traces of haemolysed and non-haemolysed blood were detected in mouse urine, and these did not correlate with the progression of infection (Table S2). Thus we conclude that the progressive renal iron accumulation observed during systemic candidiasis was not mediated by kidney injury and haemolysis.

**Figure 5:**
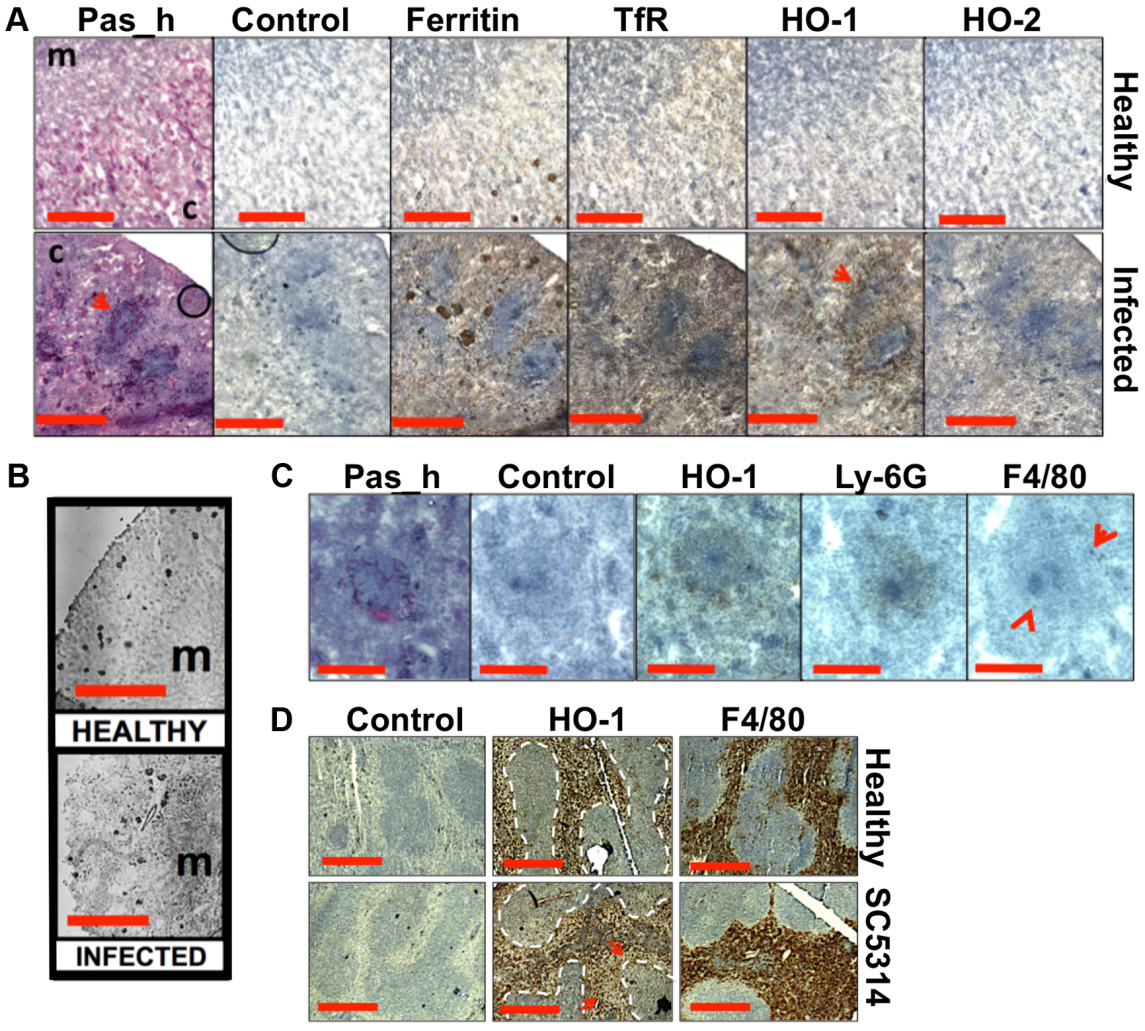
Systemic candidiasis disturbs host renal iron homeostasis and affects splenic function. **Panels A–C.** Immunohistochemical detection of iron homeostasis associated proteins in kidneys of healthy and infected animals reveals correlation with renal iron loading. Ferritin, HO-1, HO-2, and the transferrin receptor (TfR) all increase in infected kidneys in comparison to healthy controls (**A**) Ferritin is distributed outside areas occupied by *C. albicans* (**A**, arrowheads). In healthy tissue (**B, left**), ferritin content is low and mainly cortical, while increased and mainly medullary in animals with advanced candidiasis (**B, right**). HO-1 is concentrated in rings encompassing fungal lesions (**A,** arrowheads) and is produced by cell type(s) other than the Ly-6G or F4/80-producing immune cells (**C**). **Panel D**. Immunohistochemical analyses of murine spleens. Blue areas correspond to white pulp (dotted lines), embedded in the red pulp. Red pulp macrophages stain selectively with anti-HO-1 and anti-F4/80 antibodies: the stain for HO-1 is depleted in tissues from infected animals (arrows) (**D**). In all cases, the experiments represent *C. albicans* SC5314 infections in BALB/c mice. ‘Pas_h’, sections processed for histology; ‘c’, kidney cortex; ‘m’, medulla; ‘control’, experimental control with no primary antibodies. Size bars: panels **A** and **D**, 500 µm; **B**, 1000 µm; **C**, 200 µm.

Since the perfusion of infected kidneys failed to dislodge the medullary iron, we reasoned that this iron might be stored by the renal tissue. Hence the levels of renal ferritin were examined by immunohistochemistry. Normally, low levels of this major iron storage protein are present in most tissues, but ferritin abundance is up-regulated in response to iron [35]. Significantly, ferritin levels increased in infected kidneys (Fig. 5A, bottom row), primarily in the renal medulla (Fig. 5B). In contrast, the weaker ferritin signal observed in healthy kidneys localised primarily to the renal cortex (Fig. 5A, top row, and 5B). The cellular receptor for transferrin-bound iron [36] was also more abundant in infected than healthy tissue. Like ferritin, the transferrin receptor was concentrated outside fungal lesions (Fig. 5A). Taken together, our data suggest that some of the iron that accumulated in the renal medulla during disease progression was stored in ferritin complexes and some was bound by haem outside lesions.

Next we examined the spatial distribution of haem-iron extracting enzymes – haem oxygenases (HO) – during systemic candidiasis. In mammals, HO-1 is inducible and involved in red blood cell recycling in the spleen and general responses to inflammation, whereas HO-2 is expressed at constitutively low levels in most tissues [37], [38]. Immunohistochemical analyses of HO-2 revealed that its abundance was slightly increased in infected kidneys in areas outside the fungal lesions (Fig. 5A). Meanwhile HO-1 accumulated in distinct bands that surrounded fungal lesions (Fig. 5A). The external radii of HO-1 bands corresponded to the size of the immune infiltrates (Fig. 5A, bottom row, and Fig. 5C; also Figs. S1D & S6), analogous to the iron exclusion zones observed by LA-ICP MS (Fig. 2F), suggesting that HO-1 might contribute to host nutritional immunity mechanisms. Interestingly, HO-1 expressing cells did not react with anti-Ly-6G or anti-F4/80 specific antibodies, suggesting that cells other than neutrophil granulocytes and tissue macrophages mediate the exclusion zones (Fig. 5C).

We conclude that renal iron retention and accumulation during fungal infection is a dedicated, rather than random process that involves specific host proteins (Fig. 5A).

### The accumulation of renal iron elicited by fungal infection is accompanied by altered splenic function

**Figure 6:**
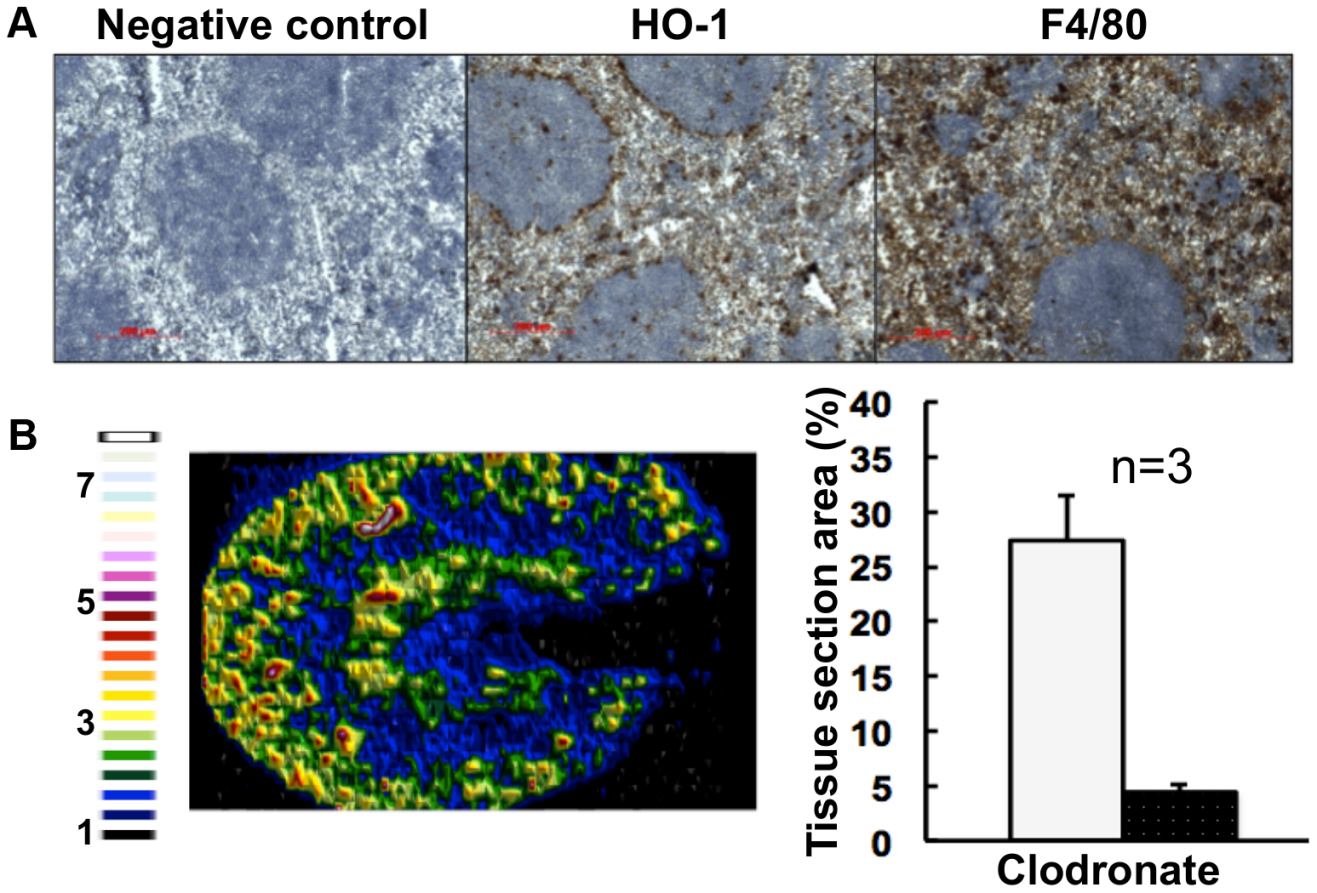
Selective chemical ablation of splenic red pulp macrophages recapitulates the renal iron accumulation phenotype in the absence of *Candida* infection. **Panel A.** Immunohistochemical confirmation of selective ablation of splenic red pulp macrophages by clodronate [41] (100 µL of 5 mg/mL liposome-encapsulated clodronate per animal): for antibodies see Fig. 5D. **Panel B.** LA-ICP MS mapping of ^56^Fe distribution reveals medullary shift of renal iron in clodronate-treated mice. Normalised ^56^Fe/^13^C ratios across kidney following 8 h clodronate treatment are presented (see panel **A**); for colour scale see Fig. 2. In the bar chart (**B**, right) bars correspond to the percentage surface area with normalized ^56^Fe/^13^C intensity≥2-fold (left, light coloured bar) and ≥3-fold above background (right, dark coloured bar): error bars, standard deviations from the mean; *n*, number of biological replicates. In all cases, the experiments represent *C. albicans* SC5314 infections in BALB/c mice. ‘Control’, experimental control with no primary antibodies. Size bars: 200 µm. Figure in **B** is representative of three biological replicates.

Most mammalian iron is present in erythrocytes and is recycled via the HO-1 synthesized by splenic red pulp macrophages. Significantly, it has been suggested that the kidney plays a major role in the retention of iron from senescent red blood cells in *HO-1^−/−^* animals whose splenic iron recycling function is severely impaired [39]. Therefore we tested the hypothesis that fungal infection perturbs splenic function. The fungal burden (CFU/g weight) in the spleen is typically a hundred-fold lower than that of the kidney in our infection model [27], and immunohistochemistry with the F4/80 macrophage marker [40] revealed that splenic red pulp macrophage numbers were not significantly reduced during infection (Fig. 5D). However, the spleens of mice with systemic candidiasis contained less HO-1 than healthy controls (Fig. 5D), indicating that splenic function was affected by the infection. Furthermore, selective partial chemical ablation of red pulp macrophages with clodronate [41] (Fig. 6A) replicated the renal iron redistribution phenotype observed during the early stages of candidiasis (Fig. 6B, Fig. S1). The clodronate treatment had no discernable impact on the F4/80 resident macrophage population in the kidney, as assessed by immunohistochemistry with specific antibodies (data not shown).

The observed changes in renal iron loading and distribution, iron regulatory and storage proteins, and splenic HO-1 function during systemic candidiasis were initially observed in BALB/c mice. Since immune responses to *C. albicans* can differ in some mouse strains, we repeated the experiments in a different mouse genetic background. All of our observations were recapitulated in C57BL/6 mice (Fig. S6). We conclude that the fungal infection affects renal and splenic iron homeostasis of the host.

### The fungal haem oxygenase pathway of iron acquisition predominates in the latter stages of lesion development

**Figure 7:**
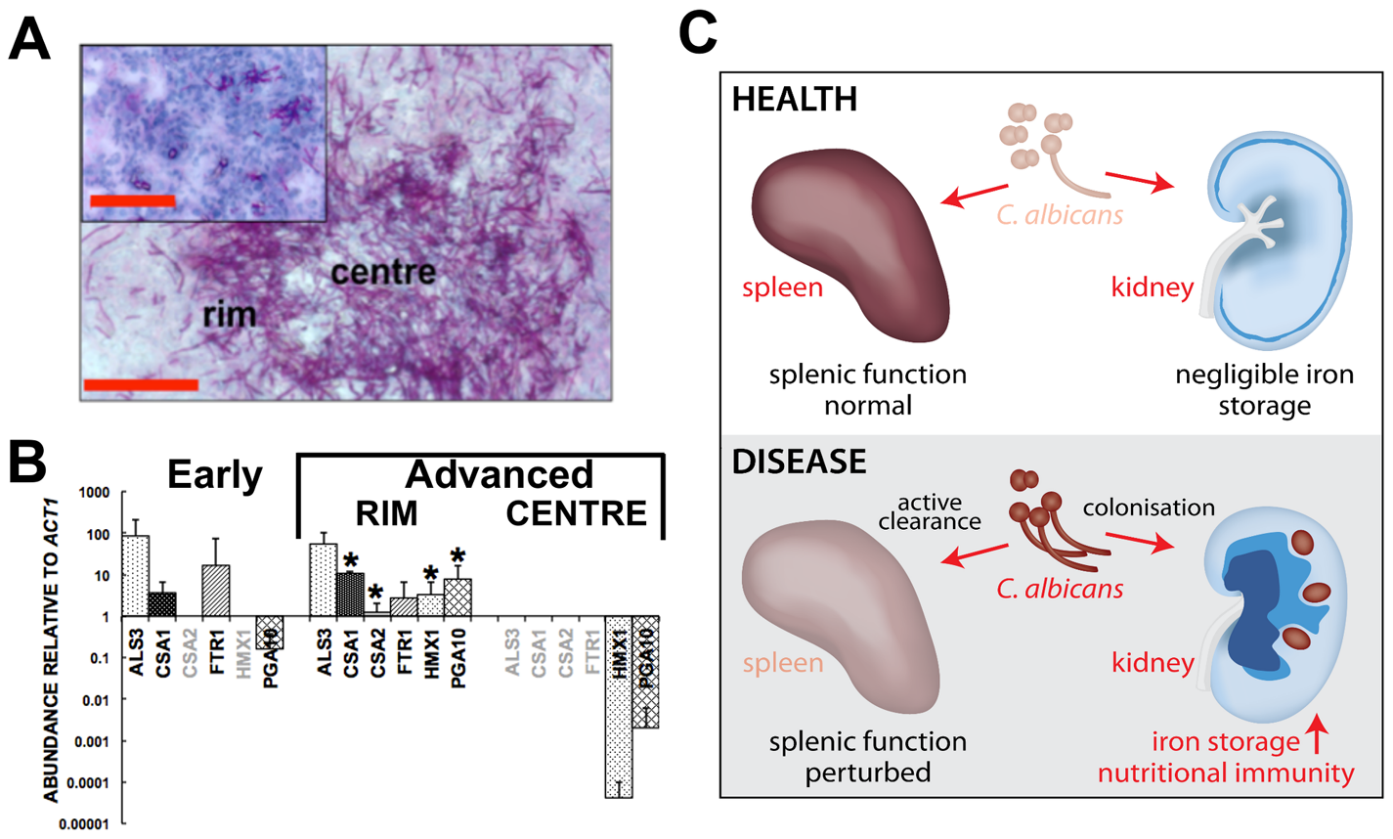
Systemic candidiasis impacts upon iron homeostasis of both the fungus and the host. **Panels A** and **B,** disturbed host iron homeostasis affects iron acquisition strategies of *C. albicans*. Fungal cells from either early (panel **A**, smaller inset) or advanced (panel **A**, larger image) stages of infection were dissected via laser capture microscopy, RNA was extracted, amplified, and abundances of the specific transcripts assessed by qRT-PCR (panel **B**). The expression of genes from the three major *C. albicans* iron acquisition pathways was assessed: (1) xenosiderophore acquisition – *SIT1* (orf19.2179) [42] (no signal, not shown); (2) reductive iron acquisition – *ALS3* (orf19.1816), *FTR1* (orf19.7219) [43]; (3) haem-iron acquisition –*CSA1* (orf19.7114), *CSA2* (orf19.3117), *PGA10* (orf19.5674), and *HMX1* (orf19.6073) [14]. The differences in gene expression patterns between the centre and rim of advanced lesions probably reflect population heterogeneity within fungal lesions [53], with the cells in the centre tightly knit and in contact with each other rather than the host, and not as metabolically active as the cells from the rim exposed to the host tissue. (**B**). The data presented are normalised to *ACT1* expression and represent means from at least three independent biological (lesion material) replicates. Greyed gene names, no transcript detection; error bars, standard deviations from the mean. Normalised transcript abundances were compared to the ‘early’ sample, differences were considered statistically significant (asterisks) for p≤0.05 in two-tailed t-test using Mann-Whitney U statistics. In panel **A**, the size bars correspond to 100 µm. **Panel C**, the effect of systemic candidiasis on the iron homeostasis of the host. During advanced candidiasis, the splenic red blood cell recycling function is perturbed presumably due to active clearance of fungal infection from that organ, while the renal involvement in host iron homeostasis increases. Blue colour intensity represents the renal iron distribution and relative concentration in the tissue, with *C. albicans* lesions represented by purple circles. See text for details.

*C. albicans* has three characterised pathways of iron acquisition: xenosiderophore-mediated, non-haem iron (reductive pathway), and haem-iron acquisition [14], [42], [43], [44]. Transcript analyses from *C. albicans* lesions harvested at various stages of development by laser capture microscopy (Fig. 7A and 7B) indicated that the fungus relies primarily on the *FTR1*-dependent reductive pathway of iron acquisition during the early stages of renal infection. However, as fungal lesions matured, *C. albicans* induced the expression of the *HMX1*-dependent haem iron acquisition pathway. Based on the regulation of these processes [14], [44], these data suggest that the fungus increasingly experiences iron limitation as the disease progresses. Indeed, we determined the average iron content across fungal lesions to be roughly half that of healthy renal cortex (24±6 µg/g versus 61±13 µg/g, respectively). This is consistent with the idea that, despite the elevated haemoglobin and haem iron loading in the renal medulla, the host limits the availability of iron to the fungal lesions in the renal cortex via nutritional immunity, akin to what has been observed for bacterial and viral infections [8].

## Discussion

Our paper addresses the question of iron homeostasis and nutritional immunity during fungal pathogenesis, an area of increasing medical importance. *In vivo* data concerning host iron homeostasis during systemic candidiasis is essentially limited to a small number of reports that focus on hepcidin and ferroportin [18]. For example, hepcidin has been reported to possess some anti-fungal properties [45]. Also, *C. albicans* infection has been shown to stimulate hepcidin production in the liver and cause a decrease in serum transferrin levels in the murine model of disseminated *Candida* infection [18]. We detected increased hepcidin levels in the kidneys of infected animals, and this was accompanied by elevated iron storage in the liver and increased HO-1 activity in that organ (Fig. 1). These results are analogous to the host response to infection by extracellular bacterial pathogens, *i.e.*, increased intracellular iron storage [4], [6].

We report here that disseminated candidiasis causes systemic perturbation of iron homeostasis by major internal organs (Fig. 1), both in organs where the fungus proliferates (kidney) and in those with lower fungal burdens (spleen). *Candida* infection triggered dramatic changes in the renal iron landscape, with the iron shifting from the renal cortex to the medulla, in a process involving specific host proteins (Figs. 2 & 5). It is known that the kidney participates in iron retention [46], but the extent of its involvement in host iron homeostasis has been far from clear. Analyses of transgenic mice lacking the main haem oxygenase, HO-1, have suggested that the kidney helps to salvage systemic iron when splenic red blood cell recycling is dysfunctional [39]. However, this relationship had not previously been demonstrated during microbial infection. Our data indicate the possibility of the iron homeostatic cross-talk between spleen and kidney under pathological conditions, when the host is suffering disseminated candidiasis. The pharmacological attenuation of splenic red pulp macrophage function by clodronate administration recapitulated the renal iron loading phenotype observed during the early stage of infection (Fig. 6). This suggested a plausible relationship between perturbed red blood cell recycling by splenic red pulp macrophages and iron accumulation in the kidney, suggesting that systemic *C. albicans* infection affects splenic function. Interestingly, Qian et al [47] reported that clodronate treatment increases the susceptibility of mice to systemic candidiasis. These authors suggested splenic involvement in the control of the infection through clearance of the pathogen from the bloodstream. Our data suggest that the effects of clodronate might instead be mediated through the exacerbation of defects in host iron homeostasis triggered by the fungal infection.

We also show that nutrient immunity operates during systemic candidiasis, further contributing to the iterative and dynamic relationship between host iron homeostasis and microbial micronutrient assimilation. Our data indicate that HO-2 promotes the assimilation of renal iron, which is then stored by ferritin in the renal medulla. Meanwhile, iron accumulation is limited locally around fungal lesions in the renal cortex via a combination of infiltrating immune cells and local increases in host HO-1 expression. We did not associate HO-1 activity with F4/80-expressing macrophages (Fig. 5C). It has been proposed that elevated HO-1 activity exerts anti-inflammatory effects through the modulatory roles of carbon monoxide (a haem breakdown product) in intracellular signalling [3], [25]. Therefore it is not inconceivable that the nutritional immunity that operates during systemic candidiasis has the dual function of depriving the pathogen of iron, and limiting inflammation at infection sites via HO-1 activity.

*C. albicans* has three characterised iron acquisition systems: (a) the *FTR1*-dependent reductive pathway [24]; (b) the *HMX1-*dependent haem-iron acquisition pathway [48]; and (c) the *SIT1-*mediated xenosiderophore acquisition pathway [14], [42]. However, it was unclear which of these pathways operates during disease onset and progression, and whether there is functional redundancy between these pathways in tissues. We show here that the fungus responds to the dramatic changes in the renal iron landscape that occur during infection by adjusting its iron acquisition strategies. We observed that *FTR1* pathway genes are expressed throughout the infection, but that the *HMX1* pathway is induced in the latter stages of infection. No expression of the *SIT1* siderophore receptor gene was detected. This is consistent with the observation that the *SIT1* pathway contributes to epithelial invasion in the presence of exogenous siderophores, presumably during dissemination from the gut [42]. On the other hand, *FTR1* appears to be essential for virulence and organ colonisation during bloodstream dissemination [24], and *HMX1* seems to be required for the maintenance of developing fungal lesions in the kidney [25]. It is conceivable that the *HMX1* pathway induction observed in latter stages of the infection was a consequence of the haemoglobin accumulation in the kidney (Fig. 2). Whatever the mechanism, *HMX1* induction could reflect the successful imposition of nutritional immunity by the host, further supported by our *in situ* iron measurements (see above). In addition, this response might be hard-wired into fungal defence mechanisms against the immune system of the host, as suggested by Navarathna and Roberts [25].

Taken together, our data show that systemic events in the host influence iron homeostasis and iron levels in major organs during fungal infection. Meanwhile, the host limits the availability of this essential micronutrient to the invading fungus through local mechanisms involving nutrient immunity (Fig. 7C). Our study provides unprecedented insights into the host-fungus interactions that revolve around iron homeostasis in the host and microbial iron assimilation during systemic fungal infection.

## Materials and Methods

### Animal experiments

*C. albicans* inocula (10^4^–10^5^ CFU/g body mass) were injected into the lateral tail veins of 6–10 week old female BALB/c or C57BL/6 mice. Infections were allowed to proceed for up to 4 days. Typically, early infections were analysed on day 2, and advanced infection on days 3–4. Advanced infection was characterised by a prevalence of large renal fungal lesions (one dimension ≥200 µm), and early infection by small fungal clusters (*circa* 60 µm in diameter, or smaller), as assessed by periodic acid/Schiff staining [49] (*vide infra*).

Harvested organs were either submerged in RNAlater solution (Qiagen, Crawley, UK) for transcript profiling, or immediately placed on dry ice and stored at −80°C. For perfusion experiments, animals were sacrificed and their blood displaced by perfusion with 1–2 mL of a sterile saline.

### *C. albicans* strains and growth conditions

*C. albicans* SC5314, AM2003/0191, and J981301 are clinical isolates [28], [30]. The construction of the *C. albicans ftr1* null mutant (Caftr1 [24]) and the *C. albicans hmx1* null mutant (DLR2 [25]) has been described. For BALB/c infections, *C. albicans* strains were grown overnight at 30°C in NGY medium [30], and for C57BL/6 infections strains were grown in Saburaud dextrose medium (Oxoid, Basingstoke, UK). The cells were grown to early stationary phase, washed and resuspended in saline, and cell density determined by hemocytometer counting before *i.v.* injection. Actual inoculum levels were confirmed by viable cell counts.

### Urinalysis

Mouse urine samples were collected, snap frozen and kept at −20°C until use. Samples were analysed using Siemens Healthcare Diagnostics dipsticks (Dipstix) according to the manufacturer's instructions.

### Tissue processing and histology

All tissues were cryosectioned in a Leica CM 1850 cryostat (Leica Biosystems, Newcastle Upon Tyne, UK). Tissue pathology was assessed with the periodic acid/Schiff reagent/hematoxylin stain [49]. Perls reagent (Polysciences, Inc., Warrington, PA) was used to visualise non-haem iron in tissues, according to the manufacturer's instructions.

### Immunohistochemistry

Specific antigens were detected in tissue sections using VECTASTAIN Elite ABC system (VectorLabs, Orton Southgate, UK) according to the manufacturer's recommendations. The following mouse-specific primary antibodies were used: FTH1 (ferritin, heavy polypeptide 1), rabbit, polyclonal (Aviva Systems Biology, San Diego, CA);F4/80, rat, monoclonal (AbDSerotec, Kidlington, UK); hepcidin-25, rabbit, polyclonal (Abcam, Cambridge, UK); HO-1, rabbit, polyclonal (Abcam, Cambridge, UK); HO-2, rabbit, polyclonal (Abcam, Cambridge, UK); Ly-6G, rabbit, polyclonal (Abcam, Cambridge, UK); transferrin receptor, rabbit, polyclonal (Abcam, Cambridge, UK). The secondary antibodies used were biotinylated horse anti-rabbit IgG (H+L) (VectorLabs, Orton Southgate, UK), or goat anti-rat IgG2b:horse radish peroxidase conjugate (AbDSerotec, Kidlington, UK), as required, using 3,3′-diaminobenzidine as the substrate (VectorLabs, Orton Southgate, UK). All images are representative of numerous replicates from at least three independent biological replicates. Brown colour indicates positive reaction; blue, no staining.

### Laser Ablation-Inductively-Coupled Plasma Mass Spectrometry (LA-ICP MS)

Sequential sections from the same tissue were prepared for LA-ICP MS, MALDI IMS (*vide supra*), and histology (*vide infra*). Cryosections (22 µm thick) were mounted on conventional glass slides and elemental distribution mapping was performed using laser ablation system (UP-213, New Wave) coupled to an Agilent 7500c ICP-MS, largely as described previously [7], [50]. Typically, the scan speed was 25 to 50 µm/s, with 80 µm laser spot size, and 20 µm spacing between the lines. Data were normalised as ^56^Fe to ^13^C intensity ratios [29] and were plotted using Microsoft Excel v14.2.4. Unless otherwise specified, the images are representative of at least two biological replicates. Quantitative iron measurements were conducted as described [51] and are averages from two biological replicates. Fungal lesion iron content was determined based on measurements from six fungal lesions. The numbers are given as µg per g dry organ weight and errors are standard deviations from the mean.

### Matrix-Assisted Laser Desorption Ionisation Time of Flight Imaging Mass Spectrometry (MALDI IMS)

Before processing, optical images of 12 µm thick tissue cryosections were acquired with NikonCoolScan, V ED slide scanner (Nikon, Kingston upon Thames, UK) for co-registration with subsequent MALDI IMS experiments. The sections were ethanol washed as per manufacturer's instructions and spray-coated (ImagePrep, BrukerDaltonics) with the appropriate matrix∶sinapic acid for native MALDI IMS (10 µg/mL) (Fluka, Dorset, UK); in-house recrystallized cyano-4-hydroxy-cinnamic acid (7 µg/mL) for peptide and haem imaging (Sigma Aldrich, Dorset, UK). For non-native imaging, tissue sections were spray-coated with sequencing-grade trypsin (Promega, Southampton, UK) solution (40 µg in 200 µL of freshly prepared 50 mM ammonium bicarbonate buffer), incubated overnight in humidified chamber (50% methanol, 38°C) and then spray-coated with matrix. MALDI TOF data were acquired with ultrafleXtreme MALDI TOF/TOF (BrukerDaltonics) at 50, 60, 70 or 100 µm resolution, in positive ion mode, and with pre-set manufacturer protocols. The data were subsequently analysed with flexImaging 2.1 and ClinProTools2.2 software (Bruker). Peptides of sufficient abundance and haem were sequenced *in situ* either from the imaged sections, or from sequential sections, using the LIFT protocol [31]. For peptide identification, the following Mascot (http://www.matrixscience.com/search_form_select.html) parameters were used for analysis of the MALDI TOF/TOF spectra: enzyme, trypsin; up to 2 missed cleavages allowed; unless otherwise indicated parental ion error ±2.0 Da; fragment ion error ±1.0 Da; preset variable modification, oxidation of methionine residues; database searched, UniProtKB/TrEMBL. Typically, peptide sequencing and identification of a given *m/z* species was performed from at least three biological replicates to confirm peptide identity.

### Identification of the major *m/z* species in native MALDI IMS spectra

To identify the protein present as a dominant peak in the average infected native MALDI IMS spectrum (14981 Da) (*vide supra*), sixteen 12 µm-thick kidney sections were collected, washed in ethanol (2×1 min 70%, 1×1 min 100%), and air dried. Proteins were extracted three times with 60∶40 ACN/0.2% TFA into a total volume of 240 µL, the extracts evaporated to dryness and residues resuspended in 0.1% TFA. 100 µL were submitted to HPLC fractionation on Brownlee Aquapore RP-300 column (C8, 7 µm, 30×2.1 mm, at 40°C), using Proteineer*fc* (BrukerDaltonics) system, operated by HyStar 3.2.44.0 software. The flow rate was 100 µL/min, with the following solvents: A, 0.1%; B, 70% ACN/0.085% TFA. Proteins were fractionated using a gradient of 5–75% solvent B in 70 min, holding the gradient for 10 min, followed by a column wash in 100% solvent B. 96×100 µL fractions were collected, dried, and resuspended in 10 µL 50% ACN/0.1% TFA. After mixing 1∶1 with matrix solution (sinapic acid, *vide supra*), 1 µL was spotted on a MALDI target plate (BrukerDaltonics). MALDI TOF spectra were automatically acquired with a native MALDI IMS method on ultrafleXtreme MALDI TOF/TOF (BrukerDaltonics). Fractions containing the *m/z* species of interest were prepared and digested with sequencing-grade trypsin (Promega, Southampton, UK) following standard protocols. This was followed by liquid chromatography/tandem mass spectrometry using BrukerDaltonics HCTultra mass spectrometer with Dionex UltiMate 3000 LC system. Finally, the acquired spectra were submitted to a Mascot search, using the following parameters: enzyme, trypsin; up to 2 missed cleavages allowed; parental ion error ±1.5 Da; fragment ion error ±0.5 Da; charge: +2 and +3; preset modifications, oxidation of methionine residues (variable), carbamidomethylation of cysteine residues (fixed); the database searched was UniProtKB/TrEMBL.

### Microtranscriptomics

Transcript profiling from laser capture microdissected material was done as described elsewhere [52] with some modifications. Briefly, a Zeiss PALM system was used for microdissection of RNAlater (Qiagen, Crawley, UK) fixed tissues. This was followed by RNA extraction and amplification with Arcturus RiboAmp HS Plus Two-Round RNA Amplification Kit (Life Technologies Ltd., Paisley, UK). Roche LightCycler 480 and Universal Probes for monocolour hydrolyses reactions were employed in qRT-PCR, according to the manufacturer's instructions. Primer pairs and specific probes are listed elsewhere [52]. For the assessment of relative abundance of *PGA10* (orf19.5674) gene transcript, the following were used: PGA10.left primer, 5′-CTGGTTGTTTGTGTGTCATGC-3′; PGA10.right primer, 5′-GTTTTTAGCAACACAGTCACCAAT-3′; probe #119 (http://www.roche-applied-science.com/sis/rtpcr/upl/index.jsp). Gene expression was normalised to *ACT1*, and differences were considered statistically significant for p≤0.05 in two-tailed t-test using the Mann-Whitney U test.

### Accession numbers

Uniprot Knowledge Base (http://www.uniprot.org) accession numbers for *C. albicans* proteins mentioned in the text: *ACT1* (orf19.5007), P14235; *ALS3* (orf19.1816), O74623; *CSA1* (orf19.7114), G1UB63; *CSA2* (orf19.3117), Q5A0X8; *FTR1* (orf19.7219), Q59ZX2; *HMX1* (orf19.6073), Q5AB97; *PGA10* (orf19.5674), Q59UP6; *SIT1* (orf19.2179), Q5A2T6.

### Ethics statement

All animal experiments were conducted in compliance with United Kingdom Home Office licenses for research on animals (project license number PPL 60/4135), and were approved by the University of Aberdeen ethical review committee. Animal experiments were minimised, and all animal experimentation was performed using approaches that minimised animal suffering and maximised our concordance with the 3Rs.

## Supporting Information

Figure S1 **Renal iron loading is not affected by fungus-associated tissue damage nor by the amount of immune infiltrates.** **A.** LA-ICP MS mapping of iron distribution in kidney sections from BALB/c mice infected with *C. albicans* AM2003/0191. Normalised ^56^Fe/^13^C ratios are presented (**A**, left), the colour scale indicates fold increases in signal intensities relative to background and is the same as in Figure 2. Histology inset (**A**, right) is representative of early infection and corresponds to the tissue imaged, with the position of fungal lesions and lesion histology given in (**B**) and (**C**), respectively. Although infections with *C. albicans* AM2003/0191 stimulate negligible immune infiltrates and elicit minimal tissue damage in comparison with SC5314 strain (compare with Fig. 5A), a relatively low fungal burden is sufficient to affect renal iron distribution (compare with Fig. 2A). **D.** Immunohistochemical detection of iron homeostasis associated proteins from kidneys of infected animals. Similarly to SC5314 infections (Fig. 5), ferritin is distributed outside of *C. albicans* lesions, and HO-1-expressing host cells cluster in tight rings around the lesions. HO-2 and transferrin receptor (TfR) increase in infected kidneys in comparison to healthy controls (compare with Fig. 5A, top row), but unlike animals with SC5314 strain-induced candidiasis, they are also detected in areas overlapping with the lesions. The images are representative of at least two biological replicates. ‘Pas_h’ denotes periodic acid/Schiff reagent/hematoxylin stain; ‘c’ refers to kidney cortex; arrows point to fungal lesions; the size bar indicates 500 µm.
(TIF)

Figure S2 **Sustained renal infection is required to trigger the accumulation of iron in the renal medulla.** LA-ICP MS mapping of iron distributions in transverse mouse kidney sections is shown. Normalised ^56^Fe/^13^C ratios are presented, and the colour scale indicates fold increases in signal intensities relative to background, as described in Fig. 2. BALB/c mice were infected with *C. albicans* strains with different defects in virulence and analysed 3–4 days post injection. *C. albicans* strains used: (1) ‘FTR1’, homozygous *ftr1* deletion mutant (Caftr1), unable to establish systemic infection [24]; (2) ‘HMX1’, homozygous *hmx1* deletion mutant (DLR2), with decreased ability to sustain renal infection [25]; (3) ‘J981301’, clinical strain with limited ability to form renal lesions in the mouse model of systemic candidiasis [30]. Histology of J981301 infected tissue is presented (bottom row, right). The data are representative for one (HMX1, J981301) or two (FTR1) independent biological replicates.
(TIF)

Figure S3 **Identification of a prominent peak in the native infected kidney proteome.** **A.** Overlay of the native MALDI imaging spectra of healthy (red) and infected (green) tissues. The spectra were averaged from two technical replicates for each condition and are representative for three biological replicates. Experimental details are given in Supporting material, and the grey bars denote peaks detected during data analysis in ClinProTools programme. The green arrow denotes the 14981 Da peak of interest, which was identified in the study **B.** Examples of MALDI TOF traces from HPLC fractions, with the peak of interest well resolved and exposed in fraction #54 (black arrow in the middle panel).
(TIF)

Figure S4 **Distribution and prevalence of HBA, HBA peptides and haem in healthy and infected tissue.** Native proteins (top row), tryptic peptides (middle row), or haem (bottom row) were mapped via MALDI IMS in the course of three separate experiments. Each experiment included kidney material from advanced candidiasis and healthy controls, rendering signal intensities comparable between tissues. In each case, *i.e.*, for HBA, HBA peptides (*m/z*1529.73 and 1819.99 ions, shown here collectively, see main text for details), and haem, the signal is more intense in the infected tissue, indicating increased concentration of the analyte in that tissue. Similarly, in all three cases, the signal is predominantly cortical in kidneys from healthy controls, and medullary in advanced infection. The data were acquired at the following laser resolutions: 100 µm (native MALDI IMS), 70 µm (tryptic MALDI IMS), 80 µm (haem imaging), and are representative of at least three independent biological replicates.
(TIF)

Figure S5 **Identification of peptides prominent in tryptic renal proteomes.** **A.** Overlay of the tryptic MALDI imaging spectra of healthy (red) and infected (green) tissues. The spectra were averaged from three biological replicates for each condition. Experimental details are given in Supporting material. **B.** LIFT fragmentation MS/MS spectra and Mascot evidence for the on-tissue sequenced tryptic peptides. Spectral prevalence of these peptides and Mascot identification parameters are given (http://www.matrixscience.com/search_form_select.html). **C.** Haem B LIFT fragmentation spectrum. All LIFT fragmentation spectra are representative of at least three independent fragmentation experiments (see Materials and Methods for details).
(TIF)

Figure S6 **Systemic candidiasis impacts upon renal iron distribution and host iron homeostasis in C57BL/6 mice.** **A–B.** LA-ICP MS mapping of iron distribution in longitudinal mouse kidney sections. Normalised ^56^Fe/^13^C ratios are presented, and the colour scale indicates fold increases in signal intensities relative to background, as described in Figure 2. As the infection progresses, iron loading increases and the iron becomes redistributed from the cortex of healthy kidneys (**A**), to the medulla in advanced infections (**B**). The data are representative for two independent biological replicates, where C57BL/6 mice were either healthy or infected with the *C. albicans* SC5314 clinical isolate. The histology inset in (**B**) (bottom) corresponds to the tissue being imaged. **C**. Immunohistochemical detection of iron homeostasis associated proteins from kidneys of healthy and infected animals. In healthy tissue (top row), ferritin content is low, while in animals with advanced candidiasis (bottom row), there is an increase in medullary ferritin. Like ferritin, HO-1, HO-2, and transferrin receptor (TfR) levels all increase as kidney infection progresses (in comparison to healthy controls). These proteins localise in areas outwith the fungal lesions, and HO-1 is concentrated in rings encompassing the lesions. **D.** Immunohistochemical detection of proteins in spleens of healthy and infected animals. Round blue areas correspond to white pulp, embedded in the red pulp. Red pulp macrophages stain selectively with anti-HO-1 and anti-F4/80 antibodies, but the stain for HO-1 is less pronounced in tissues from infected animals (arrows). Panels **C**&**D** are representative of multiple technical replicates from two independent biological replicates, with brown colour signifying a positive reaction and blue – no reaction. ‘Pas_h’, periodic acid/Schiff reagent/hematoxylin stain; ‘c’, cortex (panel C); size bars denote 500 µm in (C) and 200 µm in (D), respectively. See the main text for details.
(TIF)

Table S1 **Tryptic peptides identified from healthy and infected mouse proteomes.**
(DOCX)

Table S2 **Dipstick analysis of mouse urine.**
(DOCX)
