# Supplementary figures and images for: Fungal Iron Availability during Deep Seated Candidiasis Is Defined by a Complex Interplay Involving Systemic and Local Events
Source: PLoS Pathog. 2013 Oct 17;9(10):e1003676. doi: 10.1371/journal.ppat.1003676 (PMC3798425; doi:10.1371/journal.ppat.1003676)

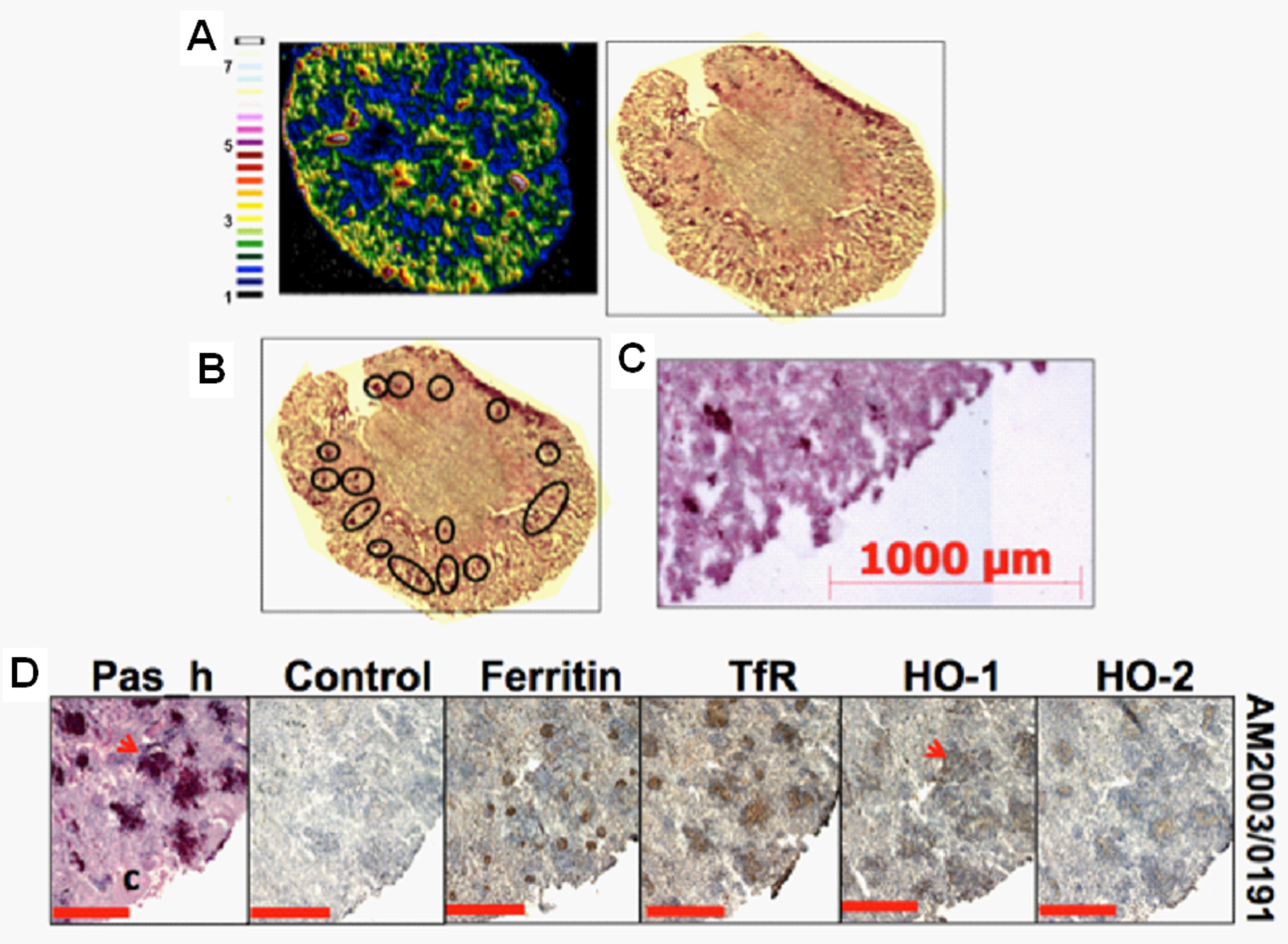

Supplement: Figure S1 — Renal iron loading is not affected by fungus-associated tissue damage nor by the amount of immune infiltrates. A. LA-ICP MS mapping of iron distribution in kidney sections from BALB/c mice infected with C. albicans AM2003/0191. Normalised 56Fe/13C ratios are presented (A, left), the colour scale indicates fold increases in signal intensities relative to background and is the same as in Figure 2. Histology inset (A, right) is representative of early infection and corresponds to the tissue imaged, with the position of fungal lesions and lesion histology given in (B) and (C), respectively. Although infections with C. albicans AM2003/0191 stimulate negligible immune infiltrates and elicit minimal tissue damage in comparison with SC5314 strain (compare with Fig. 5A), a relatively low fungal burden is sufficient to affect renal iron distribution (compare with Fig. 2A). D. Immunohistochemical detection of iron homeostasis associated proteins from kidneys of infected animals. Similarly to SC5314 infections (Fig. 5), ferritin is distributed outside of C. albicans lesions, and HO-1-expressing host cells cluster in tight rings around the lesions. HO-2 and transferrin receptor (TfR) increase in infected kidneys in comparison to healthy controls (compare with Fig. 5A, top row), but unlike animals with SC5314 strain-induced candidiasis, they are also detected in areas overlapping with the lesions. The images are representative of at least two biological replicates. ‘Pas_h’ denotes periodic acid/Schiff reagent/hematoxylin stain; ‘c’ refers to kidney cortex; arrows point to fungal lesions; the size bar indicates 500 µm. (TIF) [file ppat.1003676.s001.tif]

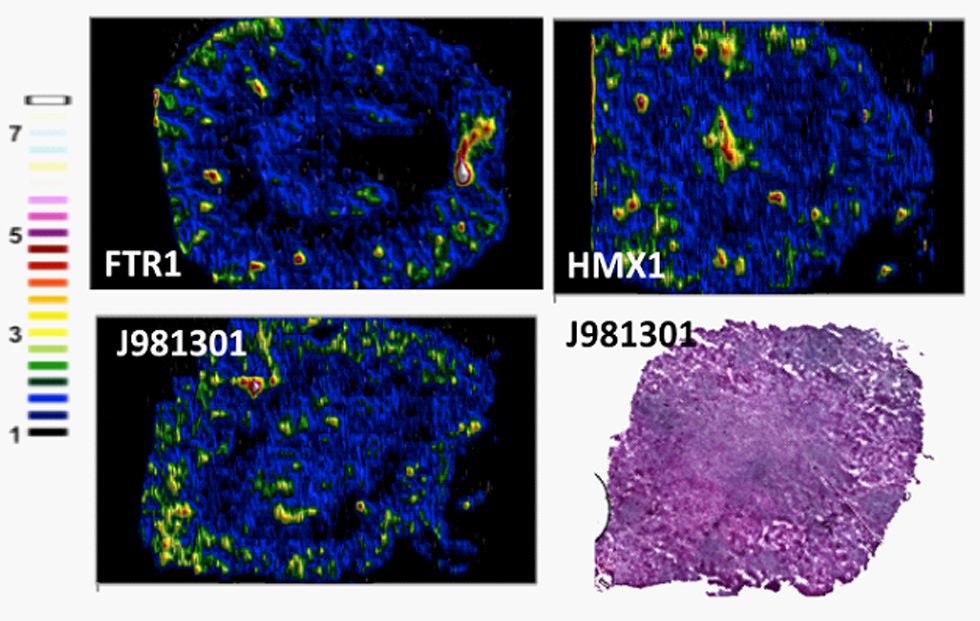

Supplement: Figure S2 — Sustained renal infection is required to trigger the accumulation of iron in the renal medulla. LA-ICP MS mapping of iron distributions in transverse mouse kidney sections is shown. Normalised 56Fe/13C ratios are presented, and the colour scale indicates fold increases in signal intensities relative to background, as described in Fig. 2. BALB/c mice were infected with C. albicans strains with different defects in virulence and analysed 3–4 days post injection. C. albicans strains used: (1) ‘FTR1’, homozygous ftr1 deletion mutant (Caftr1), unable to establish systemic infection [24]; (2) ‘HMX1’, homozygous hmx1 deletion mutant (DLR2), with decreased ability to sustain renal infection [25]; (3) ‘J981301’, clinical strain with limited ability to form renal lesions in the mouse model of systemic candidiasis [30]. Histology of J981301 infected tissue is presented (bottom row, right). The data are representative for one (HMX1, J981301) or two (FTR1) independent biological replicates. (TIF) [file ppat.1003676.s002.tif]

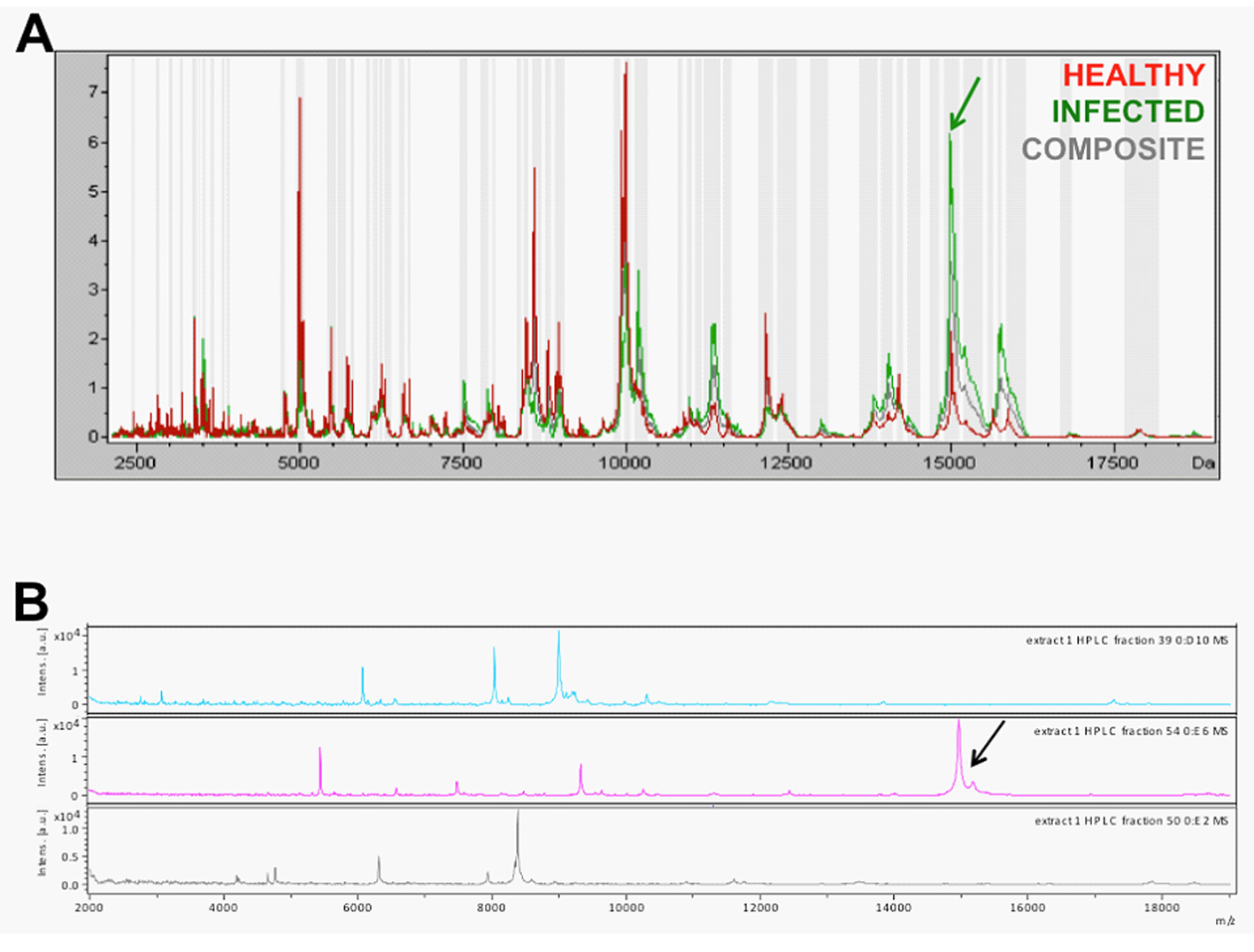

Supplement: Figure S3 — Identification of a prominent peak in the native infected kidney proteome. A. Overlay of the native MALDI imaging spectra of healthy (red) and infected (green) tissues. The spectra were averaged from two technical replicates for each condition and are representative for three biological replicates. Experimental details are given in Supporting material, and the grey bars denote peaks detected during data analysis in ClinProTools programme. The green arrow denotes the 14981 Da peak of interest, which was identified in the study B. Examples of MALDI TOF traces from HPLC fractions, with the peak of interest well resolved and exposed in fraction #54 (black arrow in the middle panel). (TIF) [file ppat.1003676.s003.tif]

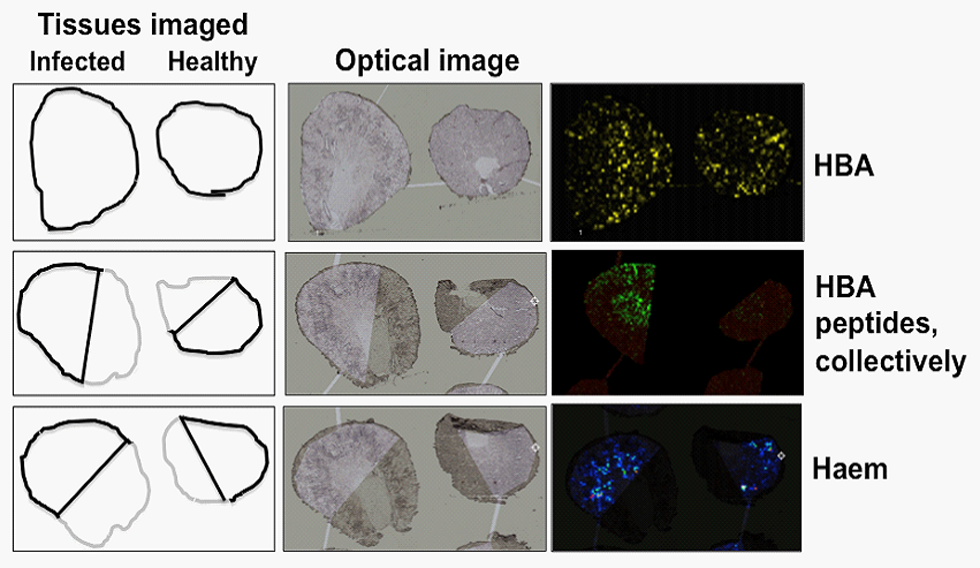

Supplement: Figure S4 — Distribution and prevalence of HBA, HBA peptides and haem in healthy and infected tissue. Native proteins (top row), tryptic peptides (middle row), or haem (bottom row) were mapped via MALDI IMS in the course of three separate experiments. Each experiment included kidney material from advanced candidiasis and healthy controls, rendering signal intensities comparable between tissues. In each case, i.e., for HBA, HBA peptides (m/z1529.73 and 1819.99 ions, shown here collectively, see main text for details), and haem, the signal is more intense in the infected tissue, indicating increased concentration of the analyte in that tissue. Similarly, in all three cases, the signal is predominantly cortical in kidneys from healthy controls, and medullary in advanced infection. The data were acquired at the following laser resolutions: 100 µm (native MALDI IMS), 70 µm (tryptic MALDI IMS), 80 µm (haem imaging), and are representative of at least three independent biological replicates. (TIF) [file ppat.1003676.s004.tif]

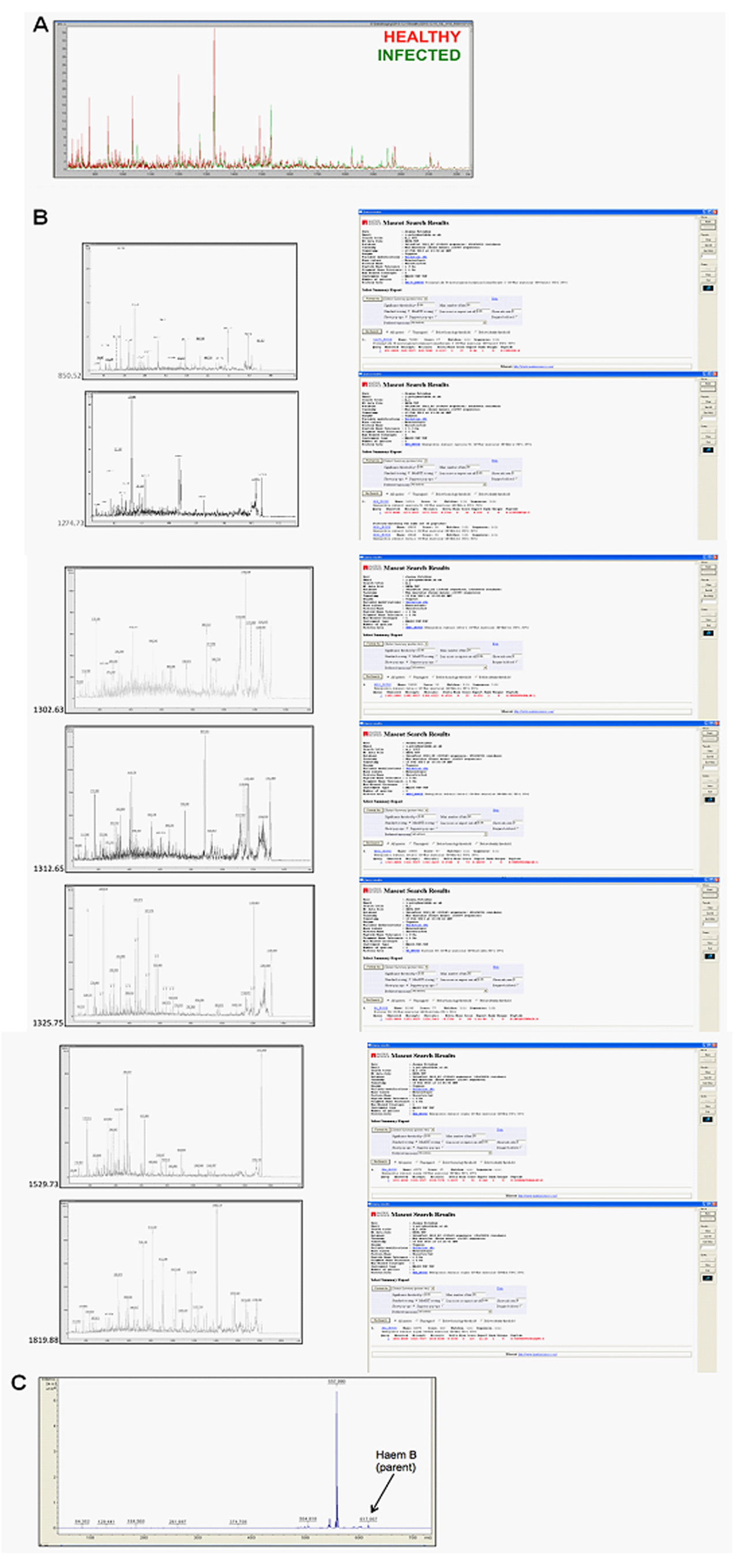

Supplement: Figure S5 — Identification of peptides prominent in tryptic renal proteomes. A. Overlay of the tryptic MALDI imaging spectra of healthy (red) and infected (green) tissues. The spectra were averaged from three biological replicates for each condition. Experimental details are given in Supporting material. B. LIFT fragmentation MS/MS spectra and Mascot evidence for the on-tissue sequenced tryptic peptides. Spectral prevalence of these peptides and Mascot identification parameters are given (http://www.matrixscience.com/search_form_select.html). C. Haem B LIFT fragmentation spectrum. All LIFT fragmentation spectra are representative of at least three independent fragmentation experiments (see Materials and Methods for details). (TIF) [file ppat.1003676.s005.tif]

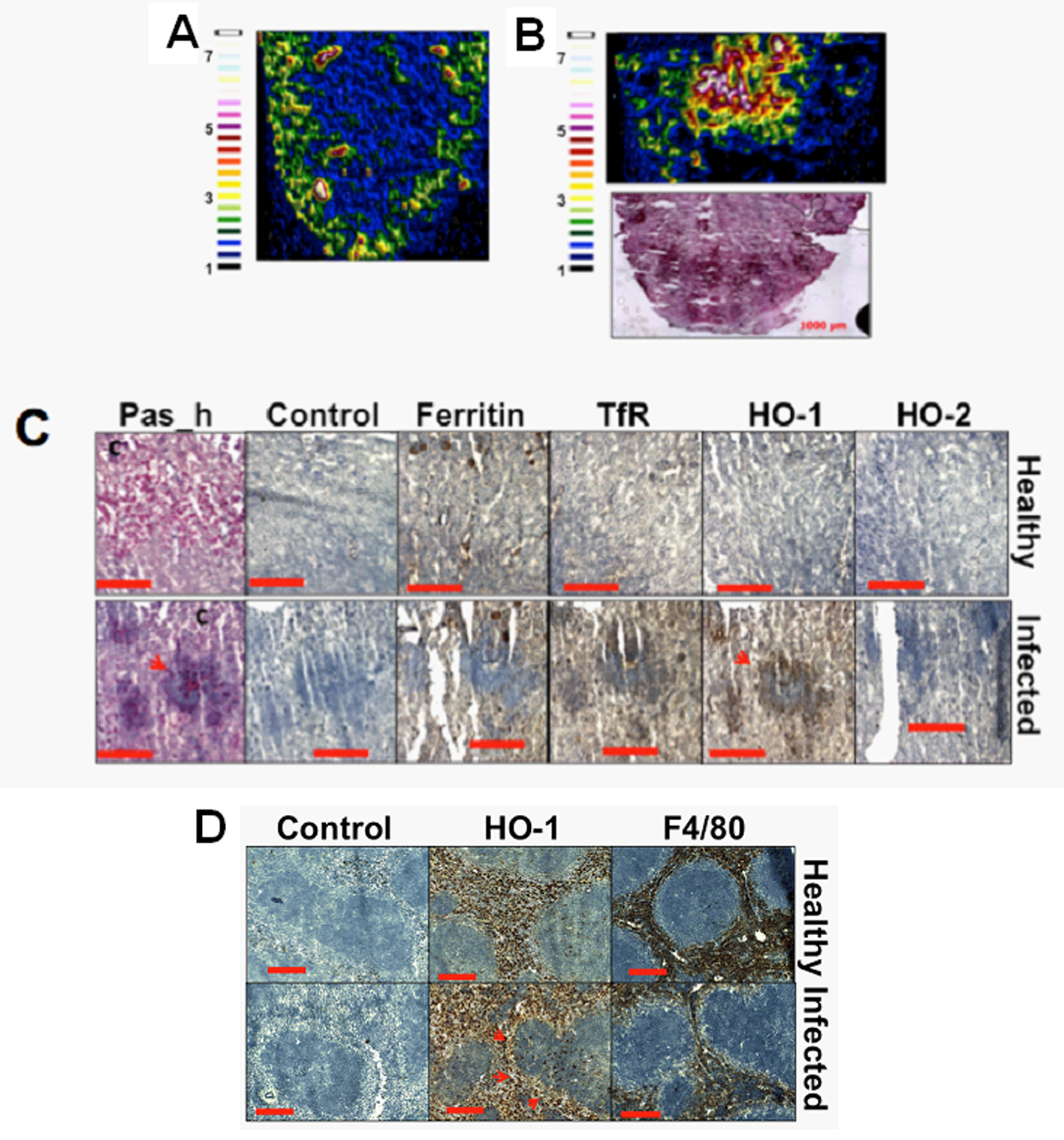

Supplement: Figure S6 — Systemic candidiasis impacts upon renal iron distribution and host iron homeostasis in C57BL/6 mice. A–B. LA-ICP MS mapping of iron distribution in longitudinal mouse kidney sections. Normalised 56Fe/13C ratios are presented, and the colour scale indicates fold increases in signal intensities relative to background, as described in Figure 2. As the infection progresses, iron loading increases and the iron becomes redistributed from the cortex of healthy kidneys (A), to the medulla in advanced infections (B). The data are representative for two independent biological replicates, where C57BL/6 mice were either healthy or infected with the C. albicans SC5314 clinical isolate. The histology inset in (B) (bottom) corresponds to the tissue being imaged. C. Immunohistochemical detection of iron homeostasis associated proteins from kidneys of healthy and infected animals. In healthy tissue (top row), ferritin content is low, while in animals with advanced candidiasis (bottom row), there is an increase in medullary ferritin. Like ferritin, HO-1, HO-2, and transferrin receptor (TfR) levels all increase as kidney infection progresses (in comparison to healthy controls). These proteins localise in areas outwith the fungal lesions, and HO-1 is concentrated in rings encompassing the lesions. D. Immunohistochemical detection of proteins in spleens of healthy and infected animals. Round blue areas correspond to white pulp, embedded in the red pulp. Red pulp macrophages stain selectively with anti-HO-1 and anti-F4/80 antibodies, but the stain for HO-1 is less pronounced in tissues from infected animals (arrows). Panels C&D are representative of multiple technical replicates from two independent biological replicates, with brown colour signifying a positive reaction and blue – no reaction. ‘Pas_h’, periodic acid/Schiff reagent/hematoxylin stain; ‘c’, cortex (panel C); size bars denote 500 µm in (C) and 200 µm in (D), respectively. See the main text for details. (TIF) [file ppat.1003676.s006.tif]
