# Supplementary material for: Fungal Iron Availability during Deep Seated Candidiasis Is Defined by a Complex Interplay Involving Systemic and Local Events
Source: PLoS Pathog. 2013 Oct 17;9(10):e1003676. doi: 10.1371/journal.ppat.1003676 (PMC3798425; doi:10.1371/journal.ppat.1003676)
Supplement: Table S1 — Tryptic peptides identified from healthy and infected mouse proteomes. (DOCX) [file ppat.1003676.s007.docx]

**Table S1. Tryptic peptides identified from healthy and infected mouse proteomes**


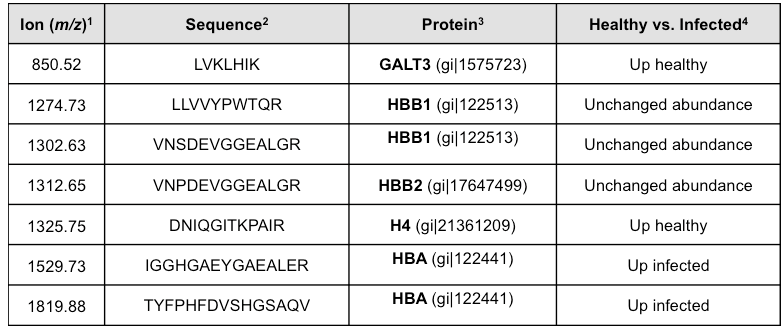


^1^Monoisotopic mass, [M+H^+^]^+1^

^2^Except for *m/z* 1819.88, all peptides were identified after LIFT sequencing directly from tissue. The fragmentation spectra are given in Supplementary Fig. S5B. For *m/z* 1819.88, MS/MS spectrum from on-tissue sequencing was not informative. The peptide was sequenced after LIFT fragmentation from a lesion-enriched tissue extract, which was submitted to in-solution digestion using standard protocols, followed by spotting onto MALDI plate and data acquisition.

^3^Proteins identified in the course of the analysis, with NCBI access numbers given in parentheses. The identification parameters are given in Figure S5.

^4^Spectral abundance of the individual peptides was determined in the course of ROC curve analysis with ClinProTools 2.2 software (Bruker), according to manufacturer’s recommendations. Increased abundance was defined when AUC≥0.85 in multiple pair-wise comparisons (2 vs. 2 biological replicates, and 3 vs. 3 biological replicates).
