# Supplementary material for: Fungal Iron Availability during Deep Seated Candidiasis Is Defined by a Complex Interplay Involving Systemic and Local Events
Source: PLoS Pathog. 2013 Oct 17;9(10):e1003676. doi: 10.1371/journal.ppat.1003676 (PMC3798425; doi:10.1371/journal.ppat.1003676)
Supplement: Table S2 — Dipstick analysis of mouse urine. (DOCX) [file ppat.1003676.s008.docx]

**Table S2. Dipstick analysis of mouse urine**


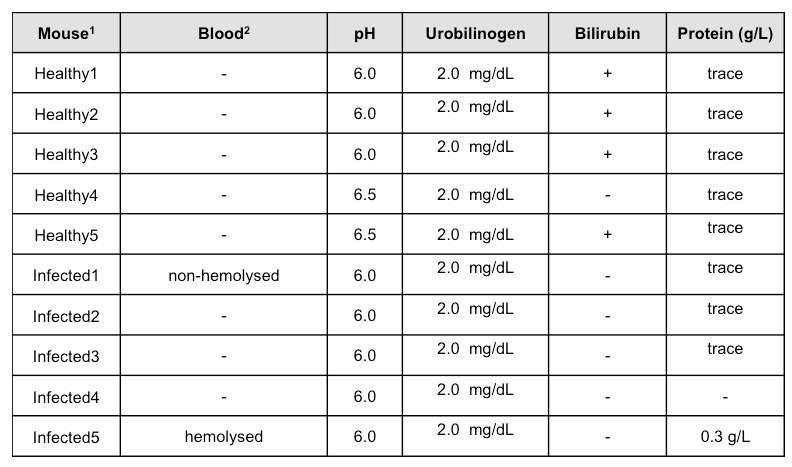


^1^Female BALB/c mice were infected with *C. albicans* SC5314 clinical isolate and urine samples collected shortly before culling. Mice ‘Infected1-3’ and ‘Infected5’ were culled on day 3 post infection; mouse ‘Infected4’ was culled on day 4 post-infection. In all instances, the infection stage was classified as intermediate to advanced, based on section histology.

^2^’Non-hemolysed’ denotes 10 erythrocytes per 1 μL, and is considered ‘trace amount’ by the test manufacturer. ‘Hemolysed’ denotes 25 erythrocytes per 1 μL.
